# Potential Application of Whole Body Vibration Exercise for Improving the Clinical Conditions of COVID-19 Infected Individuals: A Narrative Review from the World Association of Vibration Exercise Experts (WAVex) Panel

**DOI:** 10.3390/ijerph17103650

**Published:** 2020-05-22

**Authors:** Borja Sañudo, Adérito Seixas, Rainer Gloeckl, Jörn Rittweger, Rainer Rawer, Redha Taiar, Eddy A. van der Zee, Marieke J.G. van Heuvelen, Ana Cristina Lacerda, Alessandro Sartorio, Michael Bemben, Darryl Cochrane, Trentham Furness, Danúbia de Sá-Caputo, Mario Bernardo-Filho

**Affiliations:** 1 Departamento de Educación Física y Deporte, Universidad de Sevilla, 41013 Seville, Spain; bsancor@us.es; 2 Escola Superior de Saúde, Universidade Fernando Pessoa, 4200-253 Porto, Portugal; 3 Institute for Pulmonary Rehabilitation Research, Schoen Klinik Berchtesgadener Land, 83471 Schoenau am Koenigssee, Germany; rainer.gloeckl@gmx.de; 4 Department of Pulmonary Rehabilitation, Philipps–University of Marburg, German Center for Lung Research (DZL), 35037 Marburg, Germany; 5 Institute of Aerospace Medicine, German Aerospace Center (DLR), 51147 Cologne, Germany; joern.rittweger@dlr.de; 6 Department of Pediatrics and Adolescent Medicine, University of Cologne, D50931 Cologne, Germany; 7 Head of Research & Development Department, Novotec Medical GmbH & Galileo Training, 75172 Pforzheim, Germany; r.rawer@novotecmedical.de; 8 Université de Reims Champagne Ardenne, 51100 Grand Est, France; redha.taiar@univ-reims.fr; 9 Molecular Neurobiology, Groningen Institute for Evolutionary Life Sciences (GELIFES), University of Groningen, 9747 AG Groningen, The Netherlands; e.a.van.der.zee@rug.nl; 10 Department of Human Movement Sciences, University of Groningen, University Medical Center Groningen, 9713 AV Groningen, The Netherlands; m.j.g.van.heuvelen@umcg.nl; 11 Faculdade de Ciências Biológicas e da Saúde, Universidade Federal dos Vales do Jequitinhonha e Mucuri (UFVJM), Diamantina 39100-000, MG, Brazil; lacerdaacr@gmail.com; 12 Istituto Auxologico Italiano, IRCCS, Experimental Laboratory for Auxo-endocrinological Research & Division of Metabolic Diseases, 20145 Milan, Italy; sartorio@auxologico.it; 13 Department of Health and Exercise Science, University of Oklahoma, Norman, OK 73019, USA; mgbemben@ou.edu; 14 School of Sport, Exercise and Nutrition, Massey University, Private Bag 11 222, Palmerston North 4442, New Zealand; D.Cochrane@massey.ac.nz; 15 Faculty of Health Sciences, Australian Catholic University, Fitzroy, VIC 3065, Australia; Trentham.Furness@mh.org.au; 16 Laboratório de Vibrações Mecânicas, Policlínica Piquet Carneiro, Instituto de Biología Roberto Alcantara Gomes, Universidade do Estado do Rio de Janeiro, Rio de Janeiro 20950-003, Brazil; dradanubia@gmail.com (D.d.S.-C.); bernardofilhom@gmail.com (M.B.-F.); 17 Faculdade Bezerra de Araújo, Rio de Janeiro 23052-180, Brazil

**Keywords:** COVID-19, SARS-CoV-2, coronavirus, whole body vibration exercise

## Abstract

COVID-19 is a highly infectious respiratory disease which leads to several clinical conditions related to the dysfunction of the respiratory system along with other physical and psychological complaints. Severely affected patients are referred to intensive care units (ICUs), limiting their possibilities for physical exercise. Whole body vibration (WBV) exercise is a non-invasive, physical therapy, that has been suggested as part of the procedures involved with pulmonary rehabilitation, even in ICU settings. Therefore, in the current review, the World Association of Vibration Exercise Experts (WAVEX) reviewed the potential of WBV exercise as a useful and safe intervention for the management of infected individuals with COVID-19 by mitigating the inactivity-related declines in physical condition and reducing the time in ICU. Recommendations regarding the reduction of fatigue and the risk of dyspnea, the improvement of the inflammatory and redox status favoring cellular homeostasis and the overall improvement in the quality of life are provided. Finally, practical applications for the use of this paradigm leading to a better prognosis in bed bound and ICU-bound subjects is proposed.

## 1. Introduction

COVID-19 is a benign condition in 80% of symptomatic forms with about another 15% considered severe, and 5% critical, requiring resuscitation [1]. The overall lethality of symptomatic forms is estimated at 2 to 5%, depending on the age distribution of patients, their co-morbidities, and the saturation of health care systems, however, the lethality of patients with critical forms of Covid-19 has been estimated at 61% in a series of patients hospitalized in Wuhan [2] with 20% under 60 years of age [1].

COVID-19 is caused by the virus SARS-CoV-2 and results in severe stresses to the various health care systems in most countries available to combat this disease. Most infected patients have mild symptoms including fever, fatigue and cough, but in severe cases, especially elderly patients with systemic inflammatory response syndrome (SIRS), cardiovascular diseases, rheumatoid arthritis, immunodepression, cancer or chronic obstructive pulmonary disease (COPD), the disease can progress quickly to acute respiratory distress syndrome, septic shock, metabolic acidosis and coagulopathy [3]. One reason for the potential rapid deterioration associated with the disease is based on the steady accumulation of detrimental cellular and molecular changes within tissues that reduces the body’s ability to respond to stress [4]. Consequently, in some cases, the virus can also negatively impact cellular homeostasis and immunity, with some studies reporting elevations in the expression of pro-inflammatory cytokines within skeletal muscle of patients with SARS-CoV-2 infection [4].

In a recent metanalysis, Sun et al. [5] also reported that several patients with SARS-CoV-2 infection have presented with muscle soreness or fatigue as well as acute respiratory distress syndrome (ARDS), whereas diarrhea, hemoptysis, headache, sore throat, shock, and other symptoms are rare [6,7]. Suspected and confirmed cases of SARS-CoV-2 need to be treated in designated hospitals with effective isolation and protective conditions with critical cases being admitted to ICU as soon as possible. Treatment involves different approaches and recommendations generally include bed rest, with the patient being monitored for vital signs (heart rate, pulse oxygen saturation, respiratory rate, blood pressure) and given supportive treatment to ensure sufficient energy intake and water, electrolytes, and acid-base homeostasis along with other internal environment factors [8].

Considering the clinical characteristics of COVID-19 and the necessity of resting in bed, individuals are not able to perform physical activity; despite recent reports highlighting the need for these patients in maintaining regular physical activity [9]. Several authors have reported that physical activity plays an important role in the maintenance of homeostasis for individuals [10,11] and that mild to moderate intensity physical activity aids in controlling the inflammatory responses in subjects with chronic low-grade inflammation [12]. Despite these benefits, patients infected by COVID-19 cannot actively engage in any type of exercise; therefore, passive strategies such as whole-body vibration (WBV) exercise could be recommended in patients suffering from a mild COVID-19 infection after careful clinical evaluation to ensure the safety of this type of rehabilitation. WBV exercise is a non-invasive physical therapy that has even been successfully included in ICU settings [13]. These authors assessed the safety and feasibility of WBV in mechanically ventilated ICU patients and concluded that this device was both safe and feasible.

While there is evidence of the beneficial effects of WBV in numerous health outcomes in the general population [14,15], in the current review we aimed to examine the potential of WBV exercise as a useful and safe intervention for the management of infected individuals with COVID-19 in order to reduce time in ICU and/or to manage the disease sequels after recovery. This manuscript is a joint effort from members of the World Association of Vibration Exercise Experts (WAVEX), a world association of researchers interested in the potential of WBV for physical and mental health.

## 2. Effects of the WBV Exercises That Could Be Relevant to the Management of Individuals Infected with COVID-19

As reported in the previous paragraph, patients with COVID-19 typically have fever and cough and some will develop ARDS, possibly due to uncontrolled cytokine release [4]. The management of this condition in severe cases include prone positioning, lung-protective ventilation, and consideration of extracorporeal membrane oxygenation for refractory hypoxemia [16]. Therefore, in the following sections we will discuss the WBV benefits that could be relevant in the management of individuals infected with COVID-19 including: (a) the reduction of fatigue and the reduced risk of dyspnea, (b) improvements in inflammatory and redox status favoring cellular homeostasis and (c) an overall improvement in the quality of life, leading to a better prognosis in bed bound and ICU-bound subjects (Table 1).

## 3. Reduction of the Fatigue and the Risk of Dyspnea

Fatigue and dyspnea are clinical characteristics as evidenced by COVID-19 patients [17]. The fatigue is present in about 22% of infected patients [18] and, although it is still early to evaluate it in this population, a recent study suggested that 70% of ARDS survivors reported clinically significant and persistent fatigue symptoms at 6 and 12 months [19] and these are also common in patients with COPD [20] or in intensive care survivors one year after discharge [21]. Moreover, as highlighted by Neufeld et al. [19], fatigue co-occurs with impaired physical function (33% out of 711 ARDS patients) and other clinically significant symptoms, such as anxiety or depression (27%). Consequently, fatigue, weakness and negative psychological symptoms seem to be common sequelae of these conditions and this connection should be considered when considering treatment options [19]. These authors have recently shown that small increases in physical functioning status were associated with less fatigue. Although this multidimensional construct is difficult to define and may vary across a range of conditions [22], previous studies have reported that WBV exercise can, not just enhance physical status, but also manage the fatigue in various populations such as those with fibromyalgia [23]; Parkinson disease [24] or multiple sclerosis [25]. Moreover, recent studies [26] evaluated the effects of WBV (frequency 20–27 Hz) on various physical and psychological capacities in patients undergoing allogeneic hematopoietic cell transplantation (alloHCT) and reported that WBV might maintain maximum strength, functional performance, quality of life (Qol), and mitigate fatigue. In a similar fashion, Escudero-Uribe et al. [25] investigated the effects of regular exercise alone (aerobic, body weight, coordination, and balance exercises) and with the inclusion of WBV exercise (amplitude 3 mm, average frequency 4 Hz ± 1Hz/sec) on fatigue, gait pattern, mood, and quality of life in persons with relapsing-remitting multiple sclerosis (RRMS). Significant improvements in fatigue and mood were identified for both intervention groups, while gait parameters also improved significantly in the WBV group. It was concluded that combined training programs of regular exercise with WBV helps to reduce fatigue and improve mood in persons with mild to moderate RRMS. The effects of WBV exercise (amplitude 3 mm, frequency 30 Hz) was also tested in rheumatoid arthritis patients with similar improvements being reported [27]. Finally, Alentorn-Geli et al. [23] studied the effectiveness of a 6-week traditional exercise program with supplementary WBV exercise (amplitude 2 mm, frequency 30 Hz) on fibromyalgia patients (FM) and found that the WBV protocol resulted in reductions in pain and fatigue, whereas exercise alone failed to induce any improvements.

WBV exercise has also been investigated in individuals with chronic obstructive pulmonary disease (COPD). Due to lung emphysema and chronic bronchitis COPD patients suffer from severe dyspnea especially during exercise. On the other hand, research has indicated that WBV exercise does not induce dyspnea while standing on a vibration platform with knees slightly bent even though the involuntary muscle contractions that occur due to the vibrations have been shown to improve functional exercise performance as measured by the 6-min walk test in COPD patients [28]. Even dynamic activities on the vibration platform, like squat exercises, produce similar levels of dyspnea as compared to squat exercises on the floor, but with significantly greater improvements in exercise performance [29]. The current evidence suggests that WBV exercise does not induce dyspnea during training could infer that this exercise modality could be tolerated by COVID-19 patients.

In patients with stable COPD, it has been shown that WBV does not alter oxygen saturation [28,30]. In two studies reported by Furness et al. that either utilized a single session of WBV exercise consisting of five one-minute bouts of vibration (~25 Hz, ~2 mm, ~2.5 g) interspersed with five one-minute passive rest periods [24], or two sessions per week for six weeks (~25 Hz, 2 mm, ~2.5 g for WBV) [26] that neither protocol had a negative influence on oxygen saturation, an important finding when considering the use of WBV in COVID-19 patients.

## 4. Anti-Inflammatory Biomarkers Responses to WBV

In the lungs, inflammation results predominantly from tissue exposure to bacterial and viral pathogens, and/ or environmental pollutants. Excessive acute inflammation and subsequent lung injury can cause pulmonary fibrosis and impair gas exchange. Unresolved lung injury and chronic inflammation are frequently observed in acute respiratory distress syndrome, cystic fibrosis, COPD, and asthma [58,59,60]. Mitochondria are negatively affected by systemic low-grade inflammation in individuals infected with COVID-19 [4], leading to their inability to adapt to higher levels of oxidative stress and ultimately contributing to the systemic loss of muscle mass and function. Understanding the molecular basis of how systemic inflammation and exercise (e.g., WBV) influence muscle mitochondria in this patient group could provide invaluable insight into the development of exercise protocols that could maximize the beneficial adaptations of exercise.

Jawed et al. [31] explored the effects of WBV exercise (amplitude 4 mm, frequency 35 Hz) on circulating stem/progenitor cell (CPC) and cytokine levels. These authors assessed the participants (a) standing on the WBV platform, (b) performing repetitive leg squats without vibration, and (c) repetitive leg squat exercise on a vibrating platform, and reported that CPC levels increased significantly with exercise alone (i.e., repetitive leg squats) and with WBV alone in younger participants. Angiogenic CPCs increased during combined activity in younger and non-angiogenic CPCs increased with WBV alone in younger, and with exercise alone in older participants. With WBV alone, anti-inflammatory cytokine interleukin-10 increased significantly as did tumor necrosis factor-alpha and vascular endothelial growth factor, while inflammatory interleukin-6 decreased. These results suggested that WBV may have positive vascular and anti-inflammatory effects. In clinical populations, such as in COPD, Neves et al. [32] reported that WBV (amplitude 2 mm, frequency 30–40 Hz) can induce changes in inflammatory-oxidative parameters. After WBV, along with improved functional changes (e.g., 6-min walking distance, peak oxygen uptake or handgrip strength), the authors also reported improvements in inflammatory-oxidative biomarkers and white cell count. Ribeiro et al. [33] assessed the effects of a single session of WBV exercise (amplitude 4 mm, frequency 40 Hz) on inflammatory responses in a group of women diagnosed with fibromyalgia. Based on changes in levels of adipokines, soluble tumor necrosis factor receptors (sTNFr1, sTNFr2), and brain-derived neurotrophic factor (BDNF), as well as changes in oxygen consumption, heart rate, and perceived exertion (RPE), it was concluded that a single session of WBV can acutely improve the inflammatory status in patients with fibromyalgia. A similar response was observed in elderly individuals with knee osteoarthritis [34]. Plasma concentration of inflammatory markers and functional performance were assessed after squat exercises combined with WBV (amplitude 4 mm, frequency 35–40 Hz) and resulted in significantly reduced plasma concentrations of the inflammatory markers sTNFR1 and sTNFR2 accompanied by a reduction in self-reported pain.

## 5. Immune and Myokine Responses to WBV

Given the complex situation generated by the COVID-19 virus, it could be speculated that WBV is able to influence the patients’ immune system. Repeated bouts of acute exercise have been shown to enhance production of anti-inflammatory cytokines (i.e., IL-10) and myokines [61], contributing to reduced inflammation [62] as well as a reduced pro-inflammatory cytokine production [63] and an increased anti-inflammatory cytokine production [64], all of which might have an important protective role in this virus. A recent study by Song et al. [35] aimed at assessing the possible effects of WBV on immune cell differentiation and inflammatory markers reported significant increases in lymphocyte and Treg cells normally associated with improvements in the inflammation barrier function. Blanks et al. [36] also assessed the impact of WBV on the immune system in healthy participants (ages 18–45 y) (14 Hz, 2.5 mm, ~2.1 g) with 10 sets of 1 min vibration periods followed by 30 s of standing rest. The authors observed a significant increase in neutrophil percentage and increases in IL-6, a well-known myokine. This response to WBV was attributed to increased neutrophil infiltration into the muscle [65] and a resultant pro-inflammatory cytokine response [66] that would help mitigate the exercise-induced inflammatory response.

Based on the aforementioned evidence, it seems that IL-6 in response to muscle activation (i.e., WBV) has a number of anti-inflammatory benefits including increased anti-inflammatory cytokine production which could contribute to an attenuation of basal inflammation [62,67]. Blanks et al. [36] even suggested that WBV enhanced tissue IL-6 sensitivity and hypothesized, based on previous results after acute exercise [64], that this exercise paradigm would increase anti-inflammatory cytokine production (IL-10). This is consistent with a recent pilot study aimed at assessing the efficacy of WBV on inflammatory markers in individuals with chronic obstructive pulmonary disease. Participants performed WBV training (35 Hz, 2 mm, 6 × 30 s) and showed increased plasma concentrations of IL-10.

Increases in the percentage of lymphocytes in response to WBV exercise have also been observed [36,37]. Authors showed that squat exercise training with superimposed WBV might modulate T-cell-mediated immunity [37], considered a key aspect in the management of COVID-19 [4].

## 6. WBV Exercise in Bed-Bound and ICU-Bound Subjects

**Figure 1:**
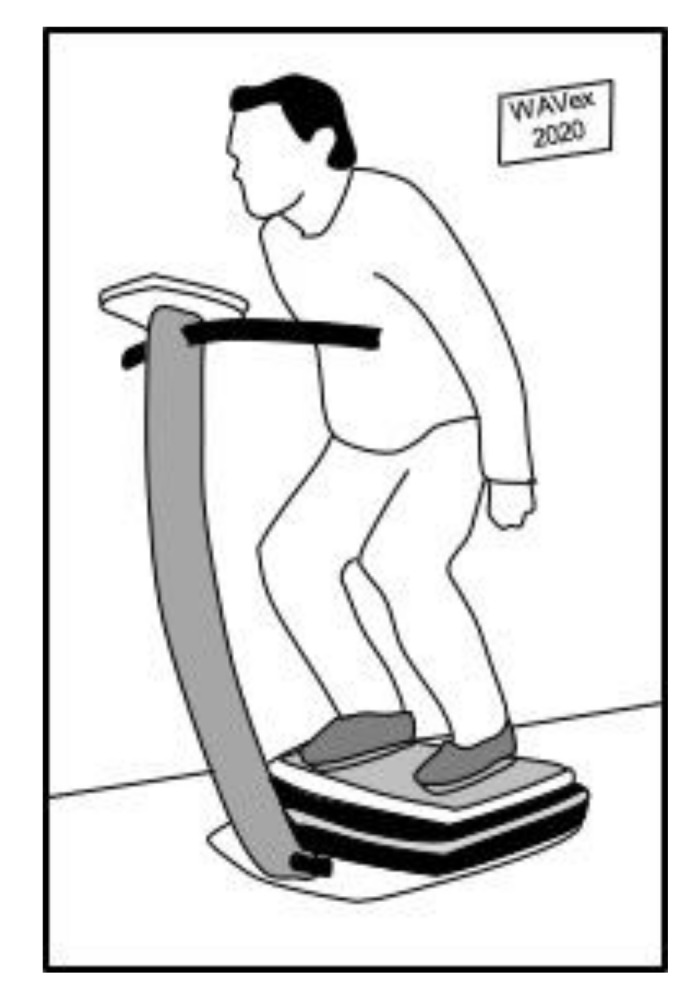
Conventional squat exercise on a WBV platform [54].

Ample evidence demonstrates the benefits of early rehabilitation in ICU-bound patients [68]. In fact, given the profound homeostatic and neuro-inflammatory processes involved during (bed-ridden) immobilization [69], one can conclude that musculoskeletal depletion is a highly detrimental side-effect of critical illness, not only with respect to successful rehabilitation after critical illness, but also for the clinical management during the active disease state. We therefore propose that ICU-bound patients are in greater need of adequate medical exercise therapy compared to ambulatory patients. However, it is obvious that traditional forms of exercise and active physiotherapy are difficult to provide in ICU patients. In the context of developing countermeasures for spaceflight that prevent physical de-conditioning, resistive WBV has been found to be a potent mode of exercise [38,70]. Given its partly passive nature, WBV is particularly useful in situations where the ability of patients to co-operate and to exercise is limited, such as in geriatrics or COPD patients hospitalized due to an acute exacerbation [39] (Figure 1). However, considering that traditional squat exercise training might be inappropriate for most COVID-19 patients, when combined with tilt-table technology, WBV can also be applied in well selected patients who are yet unable to stand by themselves, as has been powerfully demonstrated in pediatric rehabilitation [40]. Notably, the approach is also feasible in intensive care units. Technically, patients start to practice WBV in a supine position with a very small inclination, and the tilt table is then iteratively verticalized over several rehab sessions until the patients can stand freely. This approach targets all main muscle groups involved in standing and walking, and it relieves caregivers and physiotherapists from their physical labor during the period where patients are particularly unstable. Furthermore, the approach also relieves the necessity of close contacts with the patient, thus potentially reducing the spread of infectious diseases.

**Figure 2:**
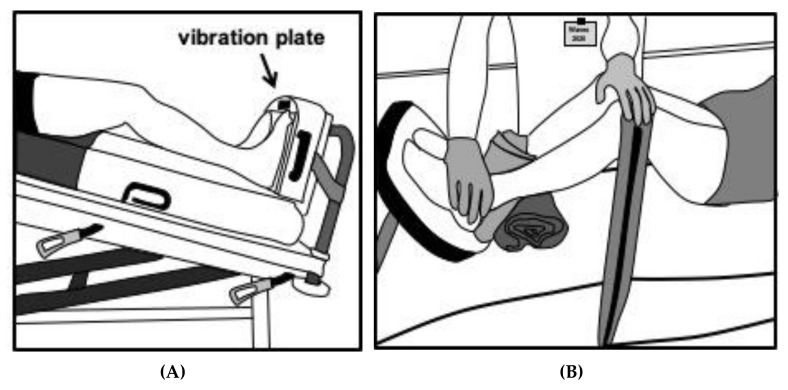
(**A**): WBV exercise in conscious but bed-bound ICU patients. Tilt ICU bed up to 30° and fix WBV platform at the end of the bed. Knees should be slightly bended for about 10°. Considerable muscle contractions at the calf and thigh muscles should be noticed by the patient (figure adapted from [44]). (**B**): WBV exercise in unconscious bed-bound ICU patients. Fix the legs with a strap to get pressure on the platform. Flex knees and hip for about 20°. Considerable muscle contractions at the calf and thigh muscles should be noticeable by a therapist (figure adapted from [13]).

On the other hand, the demands of WBV on oxygen consumption and oxygen transport are very moderate [42], as are the demands on the cardiovascular system [43]. This has not only been demonstrated in healthy men and women of all ages, but also in stable COPD patients [29] and even ICU-bound patients [13,44]. The ICU bed was either inclined up to 25° and the vibration platform was fixed on the end of the bed so that patients got some pressure with their feet on the platform (see Figure 2).

In sedated patients, the bent legs were fixed with a strap to get some pressure on the platform (see Figure 2B). In these proof-of-concept studies 1 to 3 sessions of 3 min duration were performed on a side-alternating vibration platform at a high frequency (24 Hz). In order to determine the safety of WBV exercise, vital parameters, as well as hemodynamics (e.g., oxygen saturation, respiratory rate, heart rate, blood pressure, intra-cranial pressure) were measured. Both studies demonstrated a very good feasibility of WBV exercise in ICU bed-bound patients. No clinically significant changes in vital parameters were found. No endotracheal tube, tracheal cannula, drain infusion line, ECMO-cannula, central venous catheter or dialyses catheter was dislocated during WBV [13]. Furthermore, by using electromyography a significantly increased electrical activity of the quadriceps femoris muscle during in-bed WBV exercise was found [44] indicating an important stimulus to lower extremity muscles.

In summary, the fact that WBV is a partly passive type of exercise that underpins musculoskeletal functions required for posture and locomotion, and that using a tilt-table or tilted ICU-bed in combination with WBV can safely provide critically ill patients a reduced potential of spreading the disease, making WBV a highly appealing option in the current COVID-19 pandemic.

## 7. Practical Implementation of WBV Exercise for ICU-Bound Subjects: Monitoring and Adaptation of Training Intensity

Applying a training intensity close to an individual’s limit of ability is essential to provide an effective training effect for any physical exercise [71] including WBV. Initial sessions of applying WBV as a new exercise intervention requires constant monitoring, especially in ICU applications (Table 2). In the training literature, parameters like the one-repetition maximum (1RM) are typically used as an initial measure to define a training load (for example: 80% 1RM, 10 repetitions, three sets, three times per week). Additionally, perceived exertion is often used to control training intensity, for example, if after the 3rd set, complete exhaustion is not achieved, then weights are increased for the next exercise session [71]. A similar approach could be used for adapting the intensity of WBV exercises especially in ICU conditions to promote training efficiency (maximum improvement with minimal time and risk). Since approaches like the 1RM under ICU conditions lack feasibility, then criteria like heart rate, blood pressure, SaO2 and potassium levels which are typically monitored under ICU conditions, could be used to monitor training intensity. An additional note, based on clinical observations by the authors, exercises at higher vibration frequencies in healthy individuals, the peak cardio-vascular reaction to WBV can be delayed by up to 30 s after the end of exercise. Therefore, cardiovascular response to vibration exercise for up to 90 s after the end of the exercise session should be used to evaluate individual training intensities. It should also be noted that the exercise needs to show a notable impact on these parameters in order to be effective. In other words, if no significant changes in any of the parameters mentioned above can be observed during or within 90 s after the end of the exercise session, then no training effects from the vibration exercise would be expected.

### 7.1. Application of WBV

Side-alternating WBV (SA-WBV) mimics human gait and activates muscles with an activation pattern close to human gait including muscles of the core [45]. Thus, an almost upright posture that forces the body to activate postural control and therefore allowing the vibration to activate even more muscles of the core would be ideal. Some special ICU beds in fact allow tilting angels of up to 80° which would allow such an application in principle. However, typical ICU beds allow tilting angles of 25° to 30° (Figure 2A). Considering that there is a sine relation between tilting angle and percentage loading of body weight, a 30° tilt angle is in fact equivalent to a load at 50% of body weight (Table 3). Initially, friction from the body lying on a blanket will practically decrease this value, however, application of the transmitted vibration could help to eliminate this unloading effect (see below). Effects of friction which cannot be compensated will result in less effective loading and therefore a decreased training intensity. This will automatically be compensated by increased intensity, if short term reactions as well as signs for exhaustion (Table 3), are used to control individual training intensity as proposed. The maximum possible training intensity might therefore appear to be comparably high, but this is merely reflecting the ability to compensate for friction.

Using a WBV device attached to the end of an ICU bed in combination with tilting angles of up to 30° seems to be most feasible in severe COVID-19 patients (Figure 2A). In less severe cases, after initial training effects have been achieved, a WBV device attached to a mobile tilt-table or with a standing device next to the ICU bed can also be feasible.

### 7.2. Adjusting Training Intensity

Using a tilt angle of 0° and 20° with bent legs (Figure 2B) could be used as a very low-intensity starting condition but relies, to a certain extent, on active participation of the subject or on strapping the legs down [13]. Tilting the bed with an attached WBV device (Figure 2A) can be used in patients where even less active participation is possible, and it offers an additional cardiovascular input due to partial verticalization. In practical terms, this option should be the preferred option since there is a higher potential for improved training effects. Training intensity can be controlled by various parameters beyond the tilt-angle itself, like alerting the duration, amplitude and frequency of the vibration. Once the patient is able to actively participate, intensity can further be increased by additional exercise tasks. In addition, each of the parameters in Table 4 could be used independently to significantly increase training intensity.

Based on a combination of the training parameters used by the two ICU studies focusing primarily on safety aspects [13,44] and the two studies focusing on positive intervention effects for COPD patients [29,41], as well as the practical experiences of the authors and the large body of scientific literature, the following training guidelines from Table 4 are proposed.

In principle, these guidelines aim to maximize muscle activation and at the same time, minimize the mechanical effects and are based on the following rationales: the term passive exercise which has been used to categorize vibration training is somewhat misleading, since each movement of the platform triggers a stretch-reflexed based muscle activation [46]. Hence, the term “passive” might apply to the fact that no voluntary muscle activation might be needed but the resulting exercise is caused by active muscle contraction at high levels of up to a factor of 5.5 higher when compared to quiet standing in a squatting position [46] and a factor of about 100% higher when added to intense traditional exercises like a loaded squat [47]. In order to optimize training outcomes, maximum muscle activation should therefore be the goal.

For the case of ICU application, vibration transmission within the body has positive as well as negative consequences. As a positive effect, the small movements in the hip and the torso caused by the transmission of vibration from the feet which are in contact with the vibration device, can help to eliminate effects caused by friction. As mentioned above, at a 30° tilt-angle about 50% of body weight is, in principle, applied to the vibration plate. However, friction caused by the body lying on a blanket will to a certain degree prevent the body from sliding down towards the vibration plate and therefor decreases effective loading and training intensity. The transmitted vibration can help to compensate at least a part of this effect since the transmitted movements will cause an effect similar to a vibration conveyor-system as used in industry and therefore will help to move the body towards the plate increasing the effective load. From observations a significant part of this decreased loading caused by friction can be compensated within the first 30 s of vibration application, which is one of the reasons to prefer application durations of 60 s or more. In addition, based on exercise science principles, the time under tension (TUT) of a muscle (the total active contraction time of the muscle during a set of a given exercises) for traditional exercises should be 90 to 120 s [72], which gives another rationale why a training time of 60 s is proposed as a starting point.

Negative effects of transmitted vibration could potentially be caused in the case of significant transmission of vibration to measurement sensors, infusion needles, drainage tubes and such, or to intubated patients and the Endotracheal tube. While studies examining the safety of using WBV showed no significant risk for disturbing tubes and sensors [13,44] and studies in COPD patients showed the safety and effectiveness of vibration training even when using an oxygen mask [41]. Currently, data is available concerning the use of vibration exercise in intubated patients under ICU conditions. As a consequence, the proposed guidelines aim to create high muscle activation to maximize training effects while mechanical transmissions are aimed to be as low as possible to minimize potential risks. It should be noted that research to date has shown a very low transmission of vibration from the feet to the head with 2 to 5% (side-alternating) and 6 to 14% of the amplitude created by the device under 100% loading conditions (free standing at different knee angles) [48], so transmission of the vibration stimulus to the Endotracheal tube would also be expected to be minimal.

Transmission of vibration within the human body (transmission factor) significantly depends on the type of vibration used, as well as the vibration parameters of frequency and amplitude. Side-alternating devices only transmit about half the vibration to the torso and head compared to vertical vibration devices [48]. Similarly, in-vivo measured joint forces have been shown to be 30% to 60% lower [49] in side-alternating systems. Despite the lower vibration transmissions and the lower resulting joint forces, muscle activation for the identical parameters of frequency and amplitude have been shown to be more than double in side-alternating devices compared to vertical devices [46]. Based on these data, the use of side-alternating systems to maximize muscle activation and minimize vibration transmission, especially in ICU’s, would be the preferred form of vibration.

Mechanical loading of the joints (joint-internal forces measured by artificial joint replacements with built-in force sensors) has been shown to be mainly influenced by the amplitude of the vibration. Doubling the frequency from 12.5 to 25 Hz, would in theory, result in a 4-fold increase in acceleration and consequently a 4-fold increase in joint forces, however, increases in hip-forces rose by only 10–15% and even decreased by 10% at the knee [45]. Additionally, the same study demonstrated that doubling the amplitude from 2 to 4 mm also increased hip-forces by only 10–15% but increased knee-forces by 20–25%. Along these same lines, several studies have shown that muscle activation, as measured by EMG, is significantly increased by oscillation frequency [45,46,50] with activation amplification of up to a factor of 5.5 compared to quiet standing and by about 80–100% when increasing oscillation frequency from 15 Hz to 30 Hz (Table 4).

As a consequence of the combination of the observations above, to increase training intensity it is preferable to follow a certain parameter sequence to maximize effects and minimize potential risks. The proposed sequence to increase training intensity therefore consists of:

Initial parameters: 30 to 60 s, if possible, twice a day. Then increase parameters in the following order e.g.,: Duration to 60 s (60 s to help to compensate effects of friction and to allow a TUT of 60 s per set), then tilt-angle (up to 30°) (increase proportion of body weight as high as possible using a typical ICU bed), then frequency (high effect on muscle activation but lower effect on vibration transmission), then duration above 60 s (adding cardio-vascular aspects due to longer training duration), then amplitude (adding additional vibration transmission but also additional muscle activation) and lastly, the additional exercise tasks (could also be added earlier but are unfeasible for most ICU patients).

## 8. Effects of WBV on Quality of Life

It was pointed out that COVID-19 leads to dysfunction at different levels (e.g., respiratory, physical, and psychological outcomes), with patients experiencing a serious decrement in the QoL. The effects of WBV on QoL have also been investigated and several authors reported that WBV exercise can improve the QoL of individuals with COPD [51,52,74].

## 9. Effects of WBV on Mental Conditions in COVID-19 Patients

Besides the aforementioned clinical aspects of the effect of WBV on various physiological systems, it should be mentioned that WBV also stimulates the brain, which could potentially contribute to improving cognitive function and the mental health of COVID-19 patients [53,75]. Although these aspects can be considered as secondary to the pulmonary dysfunction and pneumonia-like conditions, mental health is still important for these patients. This seems most critical during the recovery phase but keeping the brain more active during the acute phase of the disease may also accelerate subsequent full recovery. Pneumonia is known to affect the brain, including cognitive performance [76,77]. Declines in cognition after pneumonia can be caused by hypoxia [78,79], inflammation, and other mechanisms of organ dysfunction attributable to pneumonia [76]. Additionally, Davydow et al. [77] argued that immobility and lack of active exercise in patients hospitalized for pneumonia could exacerbate age-related muscle atrophy [80], and may worsen direct inflammatory, apoptotic, and hypoperfusion-mediated muscle fiber and neuronal degradation [81,82,83]. Also, proinflammatory cytokines are elevated in pneumonia-patients as well as in depressed patients [84,85], and prolonged neuroinflammation has been hypothesized to lead to late-life neurodegeneration [85].

Given that WBV activates the brain, and stimulates cognition [54,55], it could be of benefit for COVID-19 patients. Preclinical research showed that WBV induces enhanced neurotransmission [56], generates region-specific neuronal activity and stimulates hippocampal neurogenesis critical for cognition [75 and unpublished observations]. Moreover, results from previous studies suggest that WBV stimulates the cholinergic system, as well as the dopaminergic [57] and serotonergic systems enhancing mood (especially in case of mild depression). This is further corroborated by the finding that WBV, at least in preclinical studies, has the potential to reduce anxiety [53].

Taken together WBV, when used as an exercise modality, seems to affect the brain in a positive manner. This should perhaps not come as a surprise given that WBV as a form of passive exercise and reflects what is known of the positive impacts of active exercise on the brain [86]. Hence the use of WBV in seriously affected individuals with COVID-19 should also be considered in the light of cognition and mental health. Although the effect-size of the mental impact of WBV is small and its clinical relevance still debatable due to the lack of sufficient research, we consider it a bonus if WBV interventions are being performed on COVID-19 patients not (yet) capable of performing active exercise.

## 10. Limitations

This is a narrative review based on the best available knowledge of the effects of WBV in several contexts that might be extrapolated to patients with mild COVID-19 infection, but it is a theoretical approach and lacks validation in the context of COVID-19. The potential prescription and employment of WBV in selected COVID-19 infected subjects will require careful evaluation by multidisciplinary teams, asked to carefully evaluate the risk-benefit ratios, monitor the efficacy of WBV exercise, and tailor the protocols to the single subject’s clinical conditions during convalescence.

## 11. Practical Applications

Practical recommendations on how to perform WBV exercise in the hospital or on the ICU are presented in Table 4. Moreover, it is possible to suggest some specific practical applications of WBV exercise (i) for inpatients with COVID-19, that would relieve the symptoms of dyspnea, anxiety, and depression; eventually improve physical function and the QoL, reducing the time in ICU, (ii) for isolated patients, that could be conducted through educational videos, instructional manuals or remote consultation and (iii) for the improvement of post COVI-19 recovery and QoL.

## 12. Conclusions

It is expected that these findings could aid the authorities to plan a simple action like WBV exercise that could help infected individuals to attenuate the decline in physical function, improve post COVID-19 recovery and perhaps reduce time in ICU and allowing for more individuals to be treated. Moreover, these considerations could stimulate investigations involving the use of WBV exercise in in the COVID-19 patients.

## Figures and Tables

**Table 1:** Intervention parameters in the included studies.

Author	Participants and Age (Years/Months/Weeks) ± SD or [SE] or (Min–Max)	Condition	Study Design	Frequency (Hz)	Amplitude or PPD (mm)	Peak Acceleration (m/s^2^ or g)	Vibration Type/Device	Position/Exercises	Session Protocol	Intervention	Footwear
**Wollersheim 2017 [13]**	EG1: n = 12 EG2: n = 7 54 (52–59) years	Immobilized ICU patients	Clinical trial with longitudinal analysis (before, during, and after intervention)	EG1: 26 EG2: 24	2–5	No information	EG1: Synchronous vibration (Vibrosphere^®^, Promedvi: Sweden) EG2: side alternating vibration (Galileo, home-ICU®. Novotec Medical GmbH, Pforzheim, Germany)	Supine position with knees flexed at about 20°	One session	EG1: 9 × 1 min, 45 s rest EG2: 3 × 3 min	Socks
**Chang 2018 [15]**	n = 17 82.1 ± 8.2 years	Older people	Quasi-experimental, single-group, pretest-posttest design	12	3	No information	Vertical synchronous vibration (i-vib6050 model; Bodygreen, Changhua, Taiwan)	Stand on position	3-Month period, 3 sessions/week	10 × 60 s, 30 s rest	No information
**Alentorn-Geli 2008 [23]**	EG1: n = 11, 55.2 [3.4] years EG2: n = 12, 53.7 [2.7] years CG: n = 10, 59.3 [2.3] years	Fibromyalgia	RCT (2-factor mixed experimental design)	EG1: 30	EG1: 2	No information	Synchronous vibration (PowerPlate®, Power Plate North America, Inc., Northbrook, IL)	Static and dynamic lower extremities tasks (static and dynamic squat; ankle plantar-flexion with legs in extension; flexo-extension of the right leg or of the left leg; squat shifting the body weight from 1 leg to the other)	6-Week period, 2 sessions/week	3–6 × 4–18 min, 3 min rest	No information
**Corbianco 2018 [24]**	EG1: n = 10, 58.8 ± 3.9 years EG2: n = 10 56.9 ± 4.7 years	Parkinson’s disease	RCT	EG1: 26	EG1: 4	EG1: 106.64 m/s^2^	Side alternating vibration (Galileo, Med L2000, Novotec Medical GmbH, Pforzheim, Germany)	Isometric protocol in semi squat position with normalized workload (20–100% patient’s body weight, progressive increase of 5% body weight was added every week)	4-Week period, 4 sessions/week	20 × 1 min, 1 min rest	No information
**Pahl 2020 [26]**	EG: n = 18, 55 (50–63) years CG: n = 26, 56 (32–63) years	Allogeneic hematopoietic cell transplantation	RCT (subjects randomly allocated 1:1 to two parallel groups)	EG: 20–27	EG: 0–3	No information	Side alternating vibration (Galileo, Med L2000, Novotec Medical GmbH, Pforzheim, Germany)	Standing position: five exercises from a repertoire of 16 exercises for lower limbs, especially the knee extensors and flexors	180-Day period, 5 sessions/week	~20 min/session	Barefoot
**Prioreschi 2016 [27]**	EG: n = 16 CG: n = 15 EG: 51 ±10 yrs CG: 52 ±12 years	Reumatoid Arthitis Female	RCT	30	3	No information	Vertical synchronous vibration ((DKN XG 5.0, DKN Technology, California, USA)	Standing position holding on to the handlebars with knees slightly bent	12 weeks 2 sessions/week 15 min/session	EG: WBV 10 × 60 s, 30 s rest CG: normal activities	Barefoot
**Furness 2013 [28]**	n = 17 69 ± 8 years	COPD	Non-randomised, cross-over design to sham	25	2	24.7 m/s^2^	Side alternating vibration platform (Amazing Super Health, Melbourne, AUS)	Static squatting position with knees flexed at about 20°	One session	5 × 1 min, 1 min rest	Flat soled shoes
**Gloeckl 2017 [29]**	n = 10 62 ± 8 years	COPD	RCT cross-over study	26	5	No information	Side alternating vibration (Galileo, Novotec Medical, Pforzheim, Germany)	Dynamic squatting position with knees and hips at about 90–100°	One session	6 × 3 min, 10 repetitions per minute (to bend their knees 2 s concentric, 2 s eccentric, 2 s standing between each repetition)	Flat soled shoes
**Furness 2014 [30]**	n = 16 72 ± 7 years	COPD	non-randomized, cross-over design to sham	25	2	~24.7 m/s^2^	Side alternating vibration platform (Amazing Super Health, Melbourne, AUS)	53° knee flexion	6-Week period, 2 sessions/week	No information	Flat soled shoes.
**Jawed 2020 [31]**	n = 11 24 ± 1 (6-Young) 55 ± 3 (5-old) years	Healthy male subjects	Single site, within subjects, pre and post-test design, cross-over	35	4	No information	Power Plate my3 (Power Plate North America, Northbrook, IL)	EG1: standing platform vibration; EG2: repetitive leg squat exercise (no vibration); and EG3: EG1 plus EG2 (with vibration)	2 to 3-week period, one session	EG1: 8 bouts (WBV) × 60 s × 120 (rest), knees slightly bent; EG2: 8 bouts (WBV) × 60 s × 120 (rest), 90° knee flexion, 120 total repetitions of leg squats; EG3: same EG2	Barefoot
**Neves 2018 [32]**	EG: n = 10, 63.5 ± 7.8 years CG: n = 10, 63.8 ± 8.1 years	COPD	Single-blind trial with a controlled parallel design	EG: 30–40	EG: 2	EG: 1.45–2.25 g	Synchronous vibration (Fitvibe Excel Pro C, Bilzen, Belgium)	Static squatting position with knees flexed at about 30°	12-Week period, 3 sessions/week	6 × 30 s, 60 s rest	Barefoot
**Ribeiro 2018 [33]**	EG: n = 19, 52.1 [1.8] years CG: n = 19, 51.0 [1.9] years	Fibromyalgia	CT 1:1 case-control paired study (variables assessed before and immediately after one session)	EG: 40	EG: 4	No information	Synchronous vibration (Fitvibe Excel Pro C, Bilzen, Belgium)	Dynamic squatting position with knees flexed at about 10° to 60°	One session	8 × 40 s, 40 s rest (to bend their knees to 60° angle for 3 s and then to 10° angle for 3 s, over the 40 s of each series)	Barefoot
**Simão 2012 [34]**	EG1: n = 10, 75 ± 7.4 years EG2: n = 10, 69 ± 3.7 years CG: n = 11, 71 ± 5.3 years	Knee osteoarthritis	Clinical, prospective, randomized, single-blinded study	EG1: 35–40	EG1: 4	EG1: 2.00-2.61 g	Synchronous vibration (Fitvibe Excel Pro C, Bilzen, Belgium)	Dynamic squatting position with knees flexed at about 10° to 60°	12-Week period, 3 sessions/week	6–8 × 20–40 s, 20–40 s rest (to bend their knees to 60° angle for 3 s and then to 10° angle for 3 s, over each series)	Barefoot
**Song 2019 [35]**	EG1: n = 11 (hum), 22–27 years EG2: n = 10(mice), 6 wks	EG1: healthy individuals EG2: old C57BL/6 mice	Non-randomized study	EG1: 21 EG2: 13 e 17	No information	No information	Vertical vibration (Weibutexun, Jinan, China)	EG1: standing body vibration and seated for 10min in each position; EG2: no information	4-week period, 7 sessions/week	EG1: 10 min (WBV); EG2: 30 min (WBV)	No information
**Blanks 2020 [36]**	EG1: n = 11, 33 ± 4 years EG2: n = 10, 28 ± 8 years	EG1: normal weight EG2: obese	Non-randomized study	14	2.5	20.19 m/s^2^	Side alternating whole body vibration platform (RS3000, Rock Solid Wholesale, Atlantic Beach, FL, USA)	Static squat position, knee flexion (~60°) with a stable non-flexed trunk.	One session	10 bouts × 60s (WBV) × 30 s (rest)	Barefoot
**Tossige-Gomes 2012 [37]**	EG1: n = 8, 75 ± 7 years EG2: n = 10, 71 ± 4 years CG: n = 8, 72 ± 6 years	Knee osteoarthritis	Randomized controlled trial (variables assessed before and after training)	EG1: 35–40	EG1: 4	EG1: 2.78–3.26 g	Synchronous vibration (Fitvibe Excel Pro C, Bilzen, Belgium)	Dynamic squatting position with knees flexed at about 10° to 60°	12-Week period, 3 sessions/week	6–8 × 20–40 s, 20–40 s rest (to bend their knees to 60° angle for 3 s and then to 10° angle for 3 s, over each serie)	Barefoot
**Rittweger 2010 [38]**	CG: n = 10, 33.4 ± 6.6 years EG: n = 10, 32.6 ± 4.8 years	Healthy male	Randomized controlled trial	19–30	No information	No information	Side alternating vibration (Galileo Space, Novotec Medical, Pforzheim, Germany)	EG: squating exercise, heel raises, toe raises and kicks	8-week, twice daily (except for Wednesday afternoons and Sundays)	Exercises were performed rhythmically at a repetition rate of 1 in 6 s, and kicks (explosive squats with 10 s rest insertion)	No information
**Greulich 2014 [39]**	CG: n = 20, 70.4 ± 10.1 years EG: n = 20, 66.4 ± 9.93 years	COPD	Clinical trial	12–26	1.5; 2; and 3	No information	Side alternating vibration Galileo®, Novotec Medical, Pforzheim, Germany)	CG: physiotherapy program, EG: physiotherapy program plus WBV (bended knees on the Platform)	No information	3 × 2 min/day	No information
**Stark 2016 [40]**	EG1: n = 12, 8.6 ± 3.2 months EG2: n = 12, 19.4 ± 3.2 months	Cerebral palsy	Prospective, evaluator-blinded, monocenter, randomized waiting-control design with follow-up	12 or 22	2.5	0.72 g or 2.43 g	Side alternating vibration Galileo® system combined with a tilt table (Novotec Medical GmbH, Pforzheim, Germany)	Standing still or alternately squatting and standing up (using tilt table); sitting on the platform; four-point position	14-week, twice daily (10 times per week)	Ten 9-minute (3 × 3) min Feet or hands were placed at equal distance from the center of the platform	If possible the children trained without shoes, but with socks
**Gloeckl 2017 [41]**	CG: n = 37, 63 ± 9 years EG: n=37, 65 ± 8 years	COPD	Randomized controlled trial	24–26	5 PPD	No information	Side-alternating vibration platform Galileo® (Novotec Medical GmbH, Pforzheim, Germany)	Dynamic squat training, 90° and 120° Knee and hip flexion during each squat movement without holding on to anything	3-week, 3 times a week (non-consecutive days)	4 bouts × 120 s (WBV)	Flat soled shoes
**Rittweger 2001 [42]**	n = 12, 25.2 years	Healthy individuals	Non-randomized study	26	6	No information	Side alterning vibration Galileo, 2000 (Novotec Medical GmbH, Pforzheim, Germany)	Standing, squatting, and squatting with a load	One session	Exercises performed in randomized sequence for 3 min each	No information
**Hazell 2008 [43]**	EG1: n = 8, 25 ± 3.4 years EG2: n = 8, 25 ± 2.6 years	Healthy RA men	Non-randomized study	45	2	No information	Vertical vibration WAVE platform (Whole-body Advanced Vibration Exercise, Windsor, Canada)	EG1: seated next to the WBV device (passive, unloaded), 90º knee flexion EG2: semi-squat (static, loaded), 120° knee flexion	One session	EG1 and EG2: 15 repetitions of 1 min (WBV) × 1 min (rest) and 10 min of recovery (40 min of total time)	Barefoot
**Boeselt 2016 [44]**	EG1: n = 12, 41.8 ± 19.7 years EG2: n = 12, 31.3 ± 6.6 years	EG1: ICU patients EG2: healthy individuals	Non-randomized study	24	No information	No information	Side alternating vibration Galileo® (Novotec Medical, Pforzheim, Germany)	EG1 and EG2: WBV alone and WBV with a dumbbell	One session	EG1 and EG2: 3 min (WBV) × 1 min (rest) × 3 min (WBV + dumbbell)	Barefoot
**Kim 2015 [45]**	Males: n = 9, 29 ± 3.9 years Females: n = 9, 25.6 ± 3.5 years	Healthy individuals	Single-group, repeated-measure, cross-study	0, 10, 20	No information	No information	Side alternating vibration Galileo® (Novotec Medical, Pforzheim, Germany)	Three pelvic positions (neutral, anterior tilt, posterior tilt)	One session	3 × 10 s (WBV) × 10 s (rest) in each position	No information
**Ritzmann 2013 [46]**	EG1 and EG2: n = 18, 25 ± 4 years	Healthy individuals	Single-group, repeated measures, crossed-study	EG1 and EG2: 5, 10, 15, 20, 25, 30	EG 1: 2 and 4 EG2: 2	No information	EG1: Novotec Medical (Pforzheim, Germany); EG2: Power Plate. (Germany, Frankfurt am Main, Germany)		One session	EG1: side alternating vibration and EG2: synchronous vibration: 10 s (WBV) × 30 s (rest)	Barefoot
**Eckhardt 2011 [47]**	n = 14 26.0 ± 4.5 years	Physically active men	Randomizedcross-over	22	Mean 4 (feet at shoulder width)	No information	Side-alternating Galileo 900 (Novotec, Pforzheim, Germany)	Squat exercise knee bending angle 80° and additional load 10RM applied by barbell	One session	EG: WBV 5 sets of 10 squats within 30 s per set. 3 min rest between sets CC: same procedure on floor	Shoes
**Albercromby 2007 [48]**	n = 9 male 32.7± 7.0 years n = 7 female 32.7 ±8.3 years	Healthy adults	Single-group repeated measures	30	2	No information	Vertical: Powerplate Power Plate North America, Inc., Northbrook, IL) and side-alternating: Galileo 2000 (Novotec Medical, Pforzheim, Germany)	Slow dynamic squatting movement from 5° to 40° knee flexion for several	One session	Two trials for in max 15 s per condition. 60s rest between trials, 5 min rest between vibration directions	Sport socks
**Rohlmann 2014 [49]**	n = 3, 62,63,66 years	Patients fractured lumbar vertebral body, male	Repeated measures	5–25	1, 2, 4	No information	Vertical: Powerplate Pro 5. (Power Plate North America, Inc., Northbrook, IL). Side-alternating: Galileo advanced (Novotec Medical, Pforzheim, Germany)	4 postures: knees straight, knees slightly bent, knees bent at 60° and on the forefeet	One session	8 WBV trials on each plate, 12–15 s per trial, One trial 60 s. Breaks between trials 10-30s, break 5 min when changing device	No information
**Pollock 2010 [50]**	EG1: n = 12 31.3 ± 12.4 years EG2: n = 15 36 ± 12.1 years	Healthy adults	Single group repeated measures Randomized order	5–30	5.5 and 2.5	0.2–9 g	Side-alternating Galileo 2000 (Novotec Medical GmBH, Pforzheim, Germany)	Standing straight legs, without locking knees, resulting in 15.1 ± 4.8° knee flexion	One session	EC: WBV 7s for each condition (6 frequencies x 2 amplitudes) Rest 30s	Barefoot
**Braz Júnior 2015 [51]**	EG + CG: n = 11 62.91 ± 8.82	COPD, 72.7% male	Cross-over RCT	35	2 or 4 (wk1–4: 2 wk2–12: 4)	No information	Vibrating platform (MY3; Power Plate, London, UK)	Static work of the lower limbs, semi squatting position at an angle of 120°–130° with the upper limbs lightly flexed in support	12 weeks 3 sessions/week Wk 1–4: 10 min/session Wk 5–8: 15 min/session Wk 9–12: 20 min/session	EG: 1–4 wks (10 min; 30 s WBV × 60 s rest); 5-8 wks (15 min); 9-12 wks (20 min; 60 s WBV × 30 s rest) CG: no intervention	No information
**Gloeckl 2012 [52]**	EG: n = 42 CG: n = 40 EG: 64 ± 11 years CG: 65 ± 7 years	COPD, 51% female	RCT	24–26	3	No information	Side-alternating Galileo® (Novotec Medical GmbH, Pforzheim, Germany)	Squat exercises	3 weeks 3 sessions per week 3 × 3 min/session	EG: WBV 3 × 3 min CG: same exercises on floor	No information
**Boerema 2018 [53]**	EG + CG: n = 20 15 weeks	C57BI/6 mice, males	RCT	30	0.0537	0.098 g	Synchronous, 3D LEVELL R.C. Oscillator (Levell Electronics Ltd, Barnet, GB) with Shaker power amplifier	Free choice	5 weeks 5 session/week 10 minutes/session	EG: WBV CG: same procedures but without WBV	No information
**Regterschot 2014 [54]**	n = 133 20.5 ± 2.2 years	Healthy young adults, 84% female	Cross-over Short-term effects	30	0.5	No information	Vertical/Vibe 300 (Tonic Vibe, Nantes, France) with chair	Sitting	One session	EG: WBV 6 × 2 min CC: rest 6 × 2 min	Socks
**Choi 2019 [55]**	n = 18 25.3 ± 2.4 years	Healthy young male adults	Cross-over Acute effects	10, 20, 27	4	No information	Side-alternating/Galileo® Advanced Plus (Novotec Medical GmbH, Pforzheim, Germany)	Static half squat 30° flexion Standing	One session	EG: WBV 3 conditions × 2 tasks each 5 × 30 s CG: same position without WBV 3 min rest between conditions	No information
**Heesterbeek 2017 [56]**	EG + CG: n = 14 2 months	Young C57BI/6J mice, males	RCT	30	0.0537	0.098 g	Synchronous, 3D LEVELL R.C. Oscillator (Levell Electronics Ltd, Barnet, GB) with Shaker power amplifier	Free choice	5 weeks 5 session/week 10 minutes/session	EG: WBV 1 × 10 min CG: same procedures but without WBV	No information
**Zhao 2014 [57]**	EG + CG: n = 25 Body weight 25–30 g	mouse model of Parkinson’s disease C57BL mice	RCT	10 and 30	5	No information	Synchronous Platform (Columbus instruments, OH, USA)	Free choice	4 weeks 5 sessions/week 15 × 1 min/session	EG1: 5 mm/10 Hz: 15 × 1 min WBV, rest 1min EG2: 5 mm/30 Hz: 15 × 1 min WBV, rest 1 min CG1+2: same procedures without WBV	No information

SD-standard derivation; SE-standard error; Min-minimum; Max-maximum; PPD-peak-to-peak displacement; ICU-intensive care unit; wks-weeks; COPD-chronic obstructive pulmonary disease; WBV-whole body vibration; s-second; hum-humam; anim-animals; RA-recreationally active; min-minute.

**Table 2:** Training termination criteria [13].

Parameter	Value
Heart rate	<40 or >180 BPM
Systolic blood pressure	<80 mmHg or >200 mmHg;
Mean arterial blood pressure	<60 mmHg or >120 mmHg
Increase in intracerebral pressure	>20 mmHg
Oxygen saturation (SpO2)	<88%
Potassium levels	<3.0 mmol/L or >5.5 mmol/L

**Table 3:** Static loading as percentage of body weight (BW) depending on tilt angle.

Tilt Angle	% Load
10°	17% BW
20°	34% BW
30°	50% BW
60°	87% BW
80°	97% BW

**Table 4:** Parameters to alter training intensity for side-alternating and vertical whole-body vibration training devices [13,44,73].

Parameter	Value
	1 to 2
Times per day	Standard ICU bed (severe cases):
	0° tilt + 20° knee angle
	30° tilt + bent knees
Tilt Angle	Special Tilt-Table (less severe cases):
	30° to 90°
	Standing device (further increase of intensity): 90° (standing)
Frequency	Side-alternating WBV: 20 to 27 Hz Vertical WBV: 25 to 35 Hz
Duration	1 to 3 min
Number of sets	1 to 4
Amplitude (peak-to peak)	Side-alternating WBV: 1–2.5 mm (2–5 mm) Vertical WBV: 1 mm (2 mm)
Further increase of intensity by additional exercise tasks	Squatting (hip & thigh muscles)
	Heel-raises (calf muscles)
	Toe-raises (shin-muscles)
	Pelvis lifting (thigh muscles & trunk)
